# Career trends among neurosurgery residents in Pakistan: A cross-sectional study

**DOI:** 10.1097/MD.0000000000046445

**Published:** 2025-12-12

**Authors:** Bilal Khan, Muhammad Sohaib Khan, Syed Shayan Shah, Syed Jawad Ahmad, Zia Ur Rehman, Bipin Chaurasia

**Affiliations:** a Department of Neurosurgery, Lady Reading Hospital, Peshawar, Pakistan; b Department of Neurosurgery, College of Medical Science, Bharatpur, Nepal.

**Keywords:** career trends, emigration, neurosurgery, Pakistan, residents, subspecialties

## Abstract

Neurosurgery is a specialized branch of medicine that deals with the surgical treatment of conditions affecting the nervous system, including the brain, spinal cord, and peripheral nerves. Neurosurgeons undergo extensive training to diagnose and treat a wide range of neurological disorders, from brain tumors and spinal cord injuries to movement disorders and chronic pain. This nationwide survey aimed to investigate career trends, training satisfaction, and emigration intentions among neurosurgery residents in Pakistan. A prospective cross-sectional study was conducted using a structured, self-administered questionnaire disseminated via social media and email groups. One hundred twenty-two registered neurosurgery residents participated (mean age, 30.68 ± 3.50 years), with a notable gender disparity (76.2% males). Residents’ satisfaction with training improved as they progressed, with operative independence being a key factor. 82% planned to pursue a fellowship, mainly in spine and neurovascular surgery. 85.2% intended to leave Pakistan after training, favoring the UK, the Middle East, and the US. Residents in government hospitals had 7.3 times higher odds of planning emigration than those in private hospitals. Logistic regression identified private training, career uncertainty, financial pressures, and seniority as significant predictors of emigration. Latent class analysis revealed 3 challenge profiles with different emigration risks. Mediation analysis showed that satisfaction mediates the relationship between training type and emigration, particularly among junior residents. These findings highlight the urgent need for policy-level reforms to improve neurosurgical training satisfaction in Pakistan and reduce brain drain in this highly specialized field.

## 1. Introduction

Neurosurgery is a specialized branch of medicine that deals with the surgical treatment of conditions affecting the nervous system, including the brain, spinal cord, and peripheral nerves. Neurosurgeons undergo extensive training to diagnose and treat a wide range of neurological disorders, from brain tumors and spinal cord injuries to movement disorders and chronic pain. Neurological surgery is one of the most competitive and demanding specializations in medicine.^[1]^ The neurosurgery service in Pakistan began in 1951 with a spinal tumor resection surgery. Prof Bashir Ahmed, Prof Qazi, and Prof OV Jooma were the pioneers who started neurosurgery and continued to train young surgeons to provide basic services. However, developing and underdeveloped countries are facing a significant shortage of neurosurgeons, with ratios of 1 neurosurgeon per 10 million population.^[2,3]^

Neurosurgery is a demanding speciality that requires rigorous training.^[4,5]^ The training duration varies from region to region currently. In Pakistan, the College of Physicians and Surgeons Pakistan offers a nationwide training program, with a duration of 5 years. This program includes 1 year in General Surgery and 4 years in Neurosurgery, culminating in a Master of Surgery degree. The duration of the Neurosurgery program is also 5 years. In more developed countries, most institutions are willing to invest in facilities for cadaveric dissection, skill labs, and simulation technology to refine the skills of trainees. In contrast, developing countries continue to lack basic infrastructure.^[4,6]^ Sustaining good standards in neurosurgical training has been a subject of discussion in Pakistan.^[4,7]^ Due to the country’s economic conditions, most government hospitals lack the latest equipment used in neurological surgery, resulting in a deficiency in training. After completing training, a trainee can follow several paths, including joining a public or private institution as a young Neurosurgeon or pursuing subspecialty training, whether in their home country, Pakistan, or abroad. Subspecialty trainings are offered in a few hospitals in Pakistan. Different subspecialties, which include complex Spine surgery, Brain tumor surgery, Neurovascular surgery, Skull-base surgery, Endoscopic surgery, Functional neurosurgery, and Pediatric neurosurgery, are pursued. According to a survey done among neurosurgical residents, the most popular choices were spine, pediatric, and vascular fellowships.^[8]^

Several studies have examined burnout in neurosurgery residents, as well as gender disparities and prevalence in this field. However, the survey of future career trends among neurosurgery residents has not been thoroughly explored. Therefore, this area requires further research to fill the existing knowledge gap. This study aims to identify the factors that influence the career choices of young residents. So, the rationale for this study is to know the future trends among neurosurgery residents in Pakistan regarding the subspecialty training, the different foreign examinations they take during their training, their job preferences, whether to join government hospitals or private institutions and whether they want to stay in their home country or pursue a career abroad.

## 2. Materials and methods

### 2.1. Study design and ethical approval

This was a prospective, cross-sectional nationwide survey designed to evaluate career trends, training satisfaction, and emigration intent among neurosurgery residents in Pakistan. Ethical approval was obtained from the Institutional Review Board of Lady Reading Hospital, Peshawar (Approval number: 202/LRH/MTI).

### 2.2. Eligibility criteria and participant recruitment

All neurosurgery residents currently enrolled in an accredited residency training program in Pakistan were eligible to participate. Residents who had completed their training or were not part of a recognized training program were excluded. A consecutive nonprobability sampling technique was employed. Participants were recruited through electronic distribution of the survey via professional email lists and social media platforms such as WhatsApp and Facebook groups commonly used by neurosurgery trainees.

### 2.3. Survey instrument and data collection

Data were collected through a structured, self-administered questionnaire designed using Google Forms. The questionnaire included sections on demographic information, institutional affiliation, training experiences, operative exposure, perceived challenges, career aspirations, and preferences for emigration. The survey was piloted on a group of ten residents to ensure clarity and face validity; minor modifications were made based on their feedback. The final version was disseminated electronically.

### 2.4. Consent and confidentiality

Informed consent was obtained electronically at the beginning of the questionnaire. Participation was entirely voluntary, and no personally identifiable information was collected or used. All responses were anonymised, and access to raw data was restricted to the primary investigator, maintaining strict confidentiality throughout the study.

### 2.5. Data handling and statistical analysis

Survey responses were exported from Google Forms into IBM SPSS Statistics for Windows, Version 25.0 (IBM Corp., Armonk) for analysis. Descriptive statistics were used to summarize the data: continuous variables were expressed as mean ± standard deviation, while categorical variables were reported as frequencies and percentages.

Inferential statistics were employed to investigate the relationships between key variables. One-way analysis of variance (ANOVA) was performed to compare satisfaction scores across different training years. Associations between institutional type and emigration intent were assessed using Chi-square tests. To identify independent predictors of emigration, binary logistic regression analysis was conducted, with results reported as adjusted odds ratios (aORs) and 95% confidence intervals.

### 2.6. Advanced statistical methods

To explore potential mediation effects, the Hayes PROCESS macro (version 4.0) was used with 5000 bootstrap samples. Specifically, mediation analysis assessed whether training satisfaction mediated the relationship between institutional affiliation and emigration intent, with a focus on junior residents. Furthermore, latent class analysis (LCA) was performed using PROC LCA version 1.3.2 to identify distinct subgroups of residents based on reported training challenges. Model fit was evaluated using the Bayesian Information Criterion and entropy values. A *P*-value < .05 was considered statistically significant for all tests.

## 3. Results

### 3.1. Demographic and training characteristics

One hundred twenty-two registered neurosurgery residents in Pakistan participated (mean age = 30.68 ± 3.50 years). The cohort demonstrated a significant gender disparity, with males comprising 76.2% (n = 93) and females comprising 23.8% (n = 29). There was a geographic concentration in Khyber Pakhtunkhwa (41.8%, n = 51) and Punjab (35.2%, n = 43). Most trainees were in government institutions (87.7%, n = 107) and were pursuing FCPS qualifications (74.6%, n = 91).

### 3.2. Trends in resident training satisfaction

**Figure 1.**
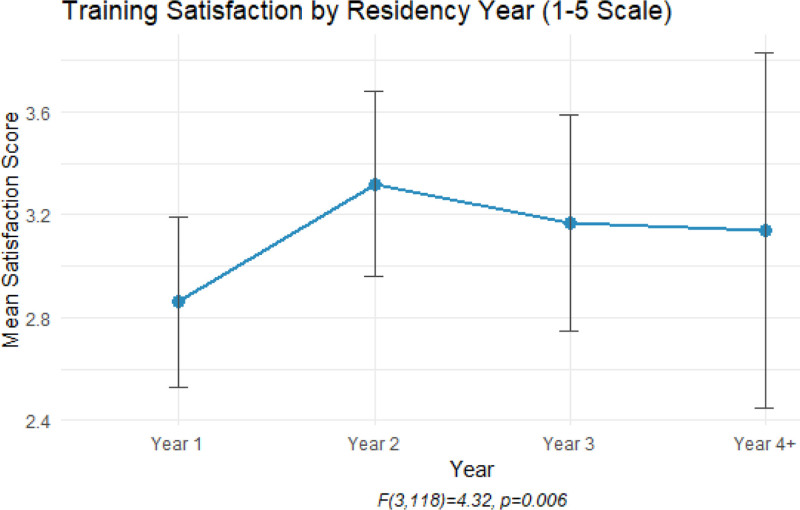
Showing training satisfaction by residency year, 2nd-year residents demonstrated the highest level of satisfaction, with 1st-year residents showing the lowest level.

The study found that residents’ satisfaction with their training significantly improved as they progressed through residency, with the highest satisfaction reported in the junior and senior years [*F*(3118) = 4.32, *P* = .006]. Satisfaction scores increased from a mean of 2.86 (1 = being very unsatisfied, 5 = very satisfied) in the first-year to around 3.14 to 3.32 in later years (Fig. 1). A key factor linked to this improvement was operative independence; residents who had more opportunities to perform surgeries reported higher satisfaction. Specifically, 63.4% of residents sometimes operated independently, 22% never did, and only 14.6% always had independence. Notably, there was a strong positive correlation (Spearman ρ = 0.72, *P* < .001) between surgical independence and residents’ technical confidence, suggesting that those with greater autonomy felt more skilled and confident in using neurosurgical instruments (Table 1).

**Table 1:** Operative independence among residents.

Operative independence	Frequency (n)	Percentage (%)
Sometimes	77	63.1
Never	27	22.1
Always	18	14.8

### 3.3. Fellowship aspiration and specialty preferences among residents

**Figure 2.**
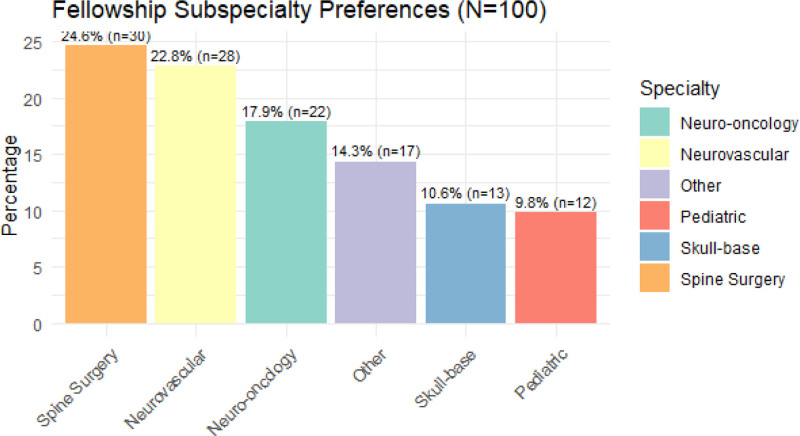
Bar graphs showing subspecialty preferences among neurosurgery residents with spine fellowship on top, followed by neurovascular.

The majority of neurosurgery residents (82%, n = 100) expressed plans to pursue a fellowship after completing their residency. The most commonly chosen subspecialties were spine surgery (24.4%), neurovascular surgery (22.8%), neuro-oncology (17.9%), skull-base surgery (10.6%), and pediatric neurosurgery (9.8%). Notably, residents intending to specialize in spine surgery reported significantly higher training satisfaction (mean score 3.87 ± 0.92) compared to those pursuing other fields [*F*(5117) = 3.14, *P* = .011], suggesting that future career interests may influence or reflect perceived quality of residency experience (Fig. 2).

### 3.4. Emigration intent and brain drain patterns

A significant majority of neurosurgery residents (85.2%, 104 out of 122) indicated plans to leave Pakistan after completing their training. Among them, 50.% (61 residents) were confident about emigrating, while 35.2% (43) were considering it. The most preferred destinations were the United Kingdom (36.9%), the Middle East (25.4%), and the United States (14.6%). Notably, residents training in government hospitals were found to have 7.3 times higher odds of planning to emigrate compared to those in private institutions (*P* < .001; 95% CI [2.1–25.9]) in the adjusted analysis, highlighting a significant disparity influenced by the type of training institution (Table 2).

**Table 2:** Emigration intent and preferred destinations (n = 122).

Category	Frequency (n)	Percentage (%)
Total planning to emigrate	104	85.2
Definite leavers	61	50.0
Considering departure	43	35.2
Top preferred destinations (n* = *104)		
United Kingdom	45	43.3
Middle East	31	29.8
United States	18	17.3

### 3.5. Multivariable predictors of emigration intent

Binary logistic regression analysis identified several significant predictors of residents’ intention to emigrate, with the model explaining 42% of the variance (Nagelkerke *R*^2^ = 0.42) and showing strong overall significance [χ^2^ (4) = 67.32, *P* < .001]. Private institute training was associated with an 86% reduction in emigration odds (adjusted odds ratio [aOR] = 0.14, 95% CI [0.04–0.48], *P* = .001) compared to government hospitals. Conversely, residents expressing career uncertainty were over 4 times more likely to consider emigration (aOR = 4.14, 95% CI [1.86–9.22], *P* < .001), and those facing financial pressures had more than double the odds (aOR = 2.36, 95% CI [1.12–4.98], *P* = .024). Additionally, being in the senior stage of training was associated with a 51% reduction in emigration intent compared to junior residents (aOR = 0.49, 95% CI [0.26–0.92], *P* = .027), as shown in Table 3. These findings highlight institutional setting, career stability, and financial factors as key drivers of emigration decisions among neurosurgery residents in Pakistan.

**Table 3:** Multivariate predictors of emigration intent (n = 122).

Predictor	aOR	95% CI	*P*-value
Institute type			
Government	1.00	–	–
Private	0.14	0.04–0.48	.001
Training stage			
Junior	1.00	–	–
Senior	0.49	0.26–0.92	.027
Primary challenge			
Career uncertainty	4.14	1.86–9.22	<.001
Financial issues	2.36	1.12–4.98	.024
Other challenges	1.02	0.42–2.48	.965

aOR = adjusted Odds ratio, CI = confidence interval.

### 3.6. LCA of residency challenges and migration intent

**Figure 3.**
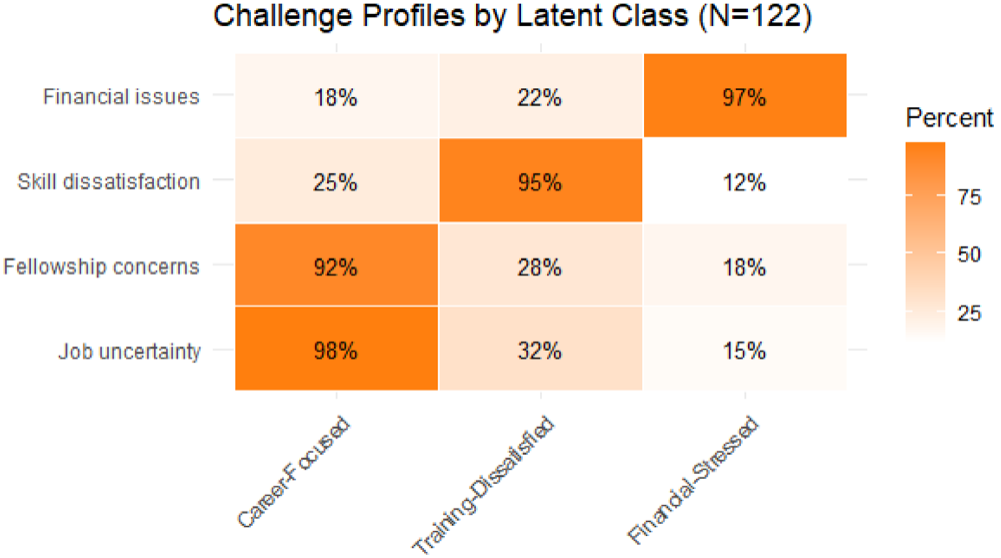
Showing the challenge profile and emigration risk.

LCA identified 3 distinct challenge profiles among neurosurgery residents, each with varying emigration risks and concerns (BIC = 1287.42, entropy = 0.89). The largest group, career-focused (51.63%), was defined by overwhelming job and fellowship uncertainty (98% and 92%, respectively) and showed the highest emigration intent at 92%. The training-dissatisfied group (27.86%) was primarily marked by dissatisfaction with their skill development and knowledge acquisition (95%), with 78% expressing intent to emigrate. The smallest group, financial-stressed (20.5%), was characterized by financial difficulties (97%) and had a 71% emigration rate (Fig. 3). Despite differing challenges, all 3 profiles demonstrated elevated emigration intent, emphasizing that diverse underlying concerns, career instability, inadequate training, and financial strain significantly contribute to brain drain among neurosurgery residents in Pakistan.

### 3.7. Mediation effect of satisfaction on emigration intent

Mediation analysis using PROCESS v4.0 (5000 bootstraps) demonstrated that overall satisfaction significantly mediated the relationship between type of training institute and emigration intent, with this effect being particularly pronounced among junior residents. The index of moderated mediation was 0.31 (95% CI [0.12, 0.53]), indicating a significant indirect effect. The strongest pathway was observed in junior residents at government hospitals, where lower satisfaction was strongly associated with higher emigration intent (β = −0.38, 95% CI [−0.62, −0.19]). This suggests that dissatisfaction during the early stages of training in public-sector institutions plays a crucial role in driving early-career emigration.

## 4. Discussion

Pakistan faces a critical neurosurgical workforce crisis, with our study revealing 85.4% of trainees plan to leave – the highest reported brain drain rate among low- and middle-income countries (LMICs). This study explored the career trajectories, training experiences, and emigration intentions of neurosurgery residents in Pakistan. By analyzing resident satisfaction, operative autonomy, fellowship preferences, and institutional affiliations, we aimed to identify the key factors driving the high rate of brain drain in this highly specialized field. While the exact number of neurosurgery residents in Pakistan is not publicly available, our sample of 122 participants is substantial. It includes trainees from all central provinces and a range of institutions, enhancing the generalizability of the findings.

A notable gender disparity was observed, with 75.6% of respondents being male. Shakir et al reported a similar trend, with 70% of neurosurgery residents in Pakistan being men.^[9]^ Shamim et al found that 33 out of 36 neurosurgery residents surveyed were male.^[6]^ This underrepresentation of women reflects global trends. In the United States, female representation in neurosurgery stood at only 9.6% as of 2021,^[10]^ with women comprising just 12% of matched residents between 2000 and 2009 and experiencing a higher attrition rate (17%) compared to males (5.3%).^[11]^ In Pakistan, career interest in neurosurgery among female medical students remains extremely low; only 2 out of 163 surveyed students considered neurosurgery, and both were male.^[12]^

A striking 85.4% of neurosurgery residents in our study expressed an intent to emigrate after training, with preferred destinations including the United Kingdom (37.4%), the Middle East (25.2%), and the United States (14.6%). Similar trends have been reported in other LMICs. Akinwumi et al noted that 63% of Nigerian residents intended to migrate to the UK, Australia, or the US.^[13]^ Tensaba et al reported that 79.4% of Nigerian medical graduates planned to emigrate due to low income, limited resources, and the desire for improved training.^[14]^

This migration trend extends beyond residency. Syed et al reported that 95% of final-year students at Aga Khan University and approximately two-thirds of those at Baqai University planned to pursue postgraduate training abroad.^[15]^

In contrast, significantly lower migration intent is seen in high-income countries. Goštautaitė et al found that only one-fifth of residents in Lithuania intended to emigrate.^[16]^ Similarly, Stummer et al in Austria and Ramos and Alves in Portugal found that fewer than half of young doctors had intentions to migrate.^[17,18]^

In our study, the primary predictors of emigration intent included career uncertainty, financial issues, fellowship concerns, dissatisfaction with skills, and being in the early years of training. Sheikh et al previously identified similar motivations among Pakistani medical students, including high salaries abroad, better training, improved quality of life, family ties, better working conditions, terrorism, physician harassment, a desire to settle abroad, intense local competition, superior management systems, peer influence, long working hours, religious considerations, parental pressure, political instability, and favoritism.^[19]^

Both public and private institutions offer postgraduate neurosurgical training in Pakistan. A higher percentage of residents from government hospitals planned to emigrate for fellowship training compared to those from private setups. Shakir et al suggested that limited exposure to cadaveric dissection, lack of simulation training, and absence of structured academic activities such as morbidity and mortality meetings in the public sector may drive this intention.^[20]^ Other LMICs report similar findings, residents in public institutions are more likely to consider emigration than those in private hospitals.^[13]^

Training satisfaction in our study steadily increased throughout residency, peaking in the later years. Operative independence emerged as a strong predictor of both satisfaction and technical confidence; residents who felt more autonomous in the operating room reported greater proficiency. In contrast, Kanju et al found that first-year residents were more satisfied than senior residents in other specialties, possibly due to initial enthusiasm and reduced responsibilities.^[21]^

Most respondents (82.1%) planned to pursue a post-residency fellowship, with spine and neurovascular surgery being the most preferred specialties. This aligns with global trends, particularly in LMICs. Gupta et al reported that among 1691 residents surveyed globally, the most preferred fellowships were spine surgery (16.04%), pediatric neurosurgery (11.18%), and cerebrovascular surgery (9.46%).^[22]^

This study is the first to provide a comprehensive, nationwide analysis of neurosurgery residents’ training satisfaction, career aspirations, and emigration intent in Pakistan. The findings underscore a significant brain drain trend, driven by career uncertainty, financial pressures, lack of operative independence, and dissatisfaction with public-sector training environments. A considerable strength of the study lies in its 100% response rate and broad geographic representation, ensuring reliable insights into the neurosurgical training landscape. The inclusion of advanced analyses, such as LCA and moderated mediation modeling, adds further depth to understanding the diverse drivers of emigration intent.

However, several limitations must be acknowledged. First, the cross-sectional design limits the ability to make causal inferences. Second, the reliance on self-reported responses may introduce reporting or recall bias; additionally, although efforts were made to include diverse institutions, some private centers may still be underrepresented. Finally, the emigration intent was measured rather than actual migration, which may evolve due to changing personal or political circumstances.

## 5. Conclusion

In conclusion, this study highlights the urgent need for policy-level reforms to improve neurosurgical training satisfaction in Pakistan, particularly within public-sector institutions. Enhancing operative exposure, structured mentorship, and financial support during early residency years may reduce emigration intent and help retain skilled neurosurgeons within the country’s healthcare system. Longitudinal tracking of these residents’ actual migration patterns could validate intent-to-action transitions and refine retention strategies.

## Acknowledgments

The authors would like to thank all the participants for their time and willingness to complete the survey, which made this study possible.

## Author contributions

**Conceptualization:** Bilal Khan.

**Data curation:** Muhammad Sohaib Khan.

**Formal analysis:** Muhammad Sohaib Khan.

**Methodology:** Bilal Khan, Zia Ur Rehman.

**Supervision:** Zia Ur Rehman.

**Visualization:** Syed Jawad Ahmad.

**Writing – original draft:** Syed Shayan Shah.

**Writing – review & editing:** Bilal Khan, Muhammad Sohaib Khan, Syed Jawad Ahmad, Zia Ur Rehman, Bipin Chaurasia.
